# Development and validation of the lifetime sense of safety scale in Kenyan adults

**DOI:** 10.1038/s43856-026-01715-2

**Published:** 2026-06-06

**Authors:** Karen Blackmon, Anne N. Nyambura, Anne Gitere, Levi A. Muyela, Catherine Bikeri, Kendi Muchungi, Catherine Ajalo, Litha M. Musili, Dina Abdalla, Rachel Maina, Edna Bosire, Lukoye Atwoli, Zul Merali, Chinedu Udeh-Momoh

**Affiliations:** 1 Aga Khan University, Brain and Mind Institute, Nairobi, Kenya; 2 Geisel School of Medicine at Dartmouth, Hanover, New Hamsphire United States of America; 3 Kenyatta University, Department of Community and Reproductive Health Nursing, Nairobi, Kenya; 4 Nairobi City County Government, Nairobi, Kenya; 5 The University of Dublin, Global Brain Health Institute, Trinity College Institute of Neuroscience, Dublin, Ireland; 6 Aga Khan University, Department of Medicine, Medical College East Africa, Nairobi, Kenya; 7 Wake Forest School of Medicine, Winston-Salem, North Carolina United States of America

**Keywords:** Limbic system, Population screening, Alzheimer's disease, Post-traumatic stress disorder, Neurodegeneration

## Abstract

**Background:**

Safety supports psychological wellbeing and potentially shapes brain aging yet existing scales overlook cumulative lifespan safety experiences.

**Methods:**

To address this gap, we developed and validated the Lifetime Sense of Safety (LSS) scale in Kenyan adults. The LSS uses visual analog scaling with a red-to-green color gradient to retrospectively rate felt safety across life stages and settings. We evaluated scale psychometrics and associations with social determinants of health and indicators of brain health.

**Results:**

The LSS shows adequate internal consistency and test-retest reliability, with two distinct but correlated factors representing childhood and adulthood safety. Women and individuals experiencing poverty report lower lifetime safety. Total LSS scores correlate negatively with stress and anxiety and positively with resilience and memory.

**Conclusions:**

The LSS is a psychometrically valid tool for evaluating cumulative lifespan safety in Kenyan adults. Longitudinal studies combining the LSS with neuroendocrine, inflammatory, and neuroimaging measures could identify mechanisms linking safety to brain health.

## Plain language summary

We developed a six-item questionnaire called the Lifetime Sense of Safety scale and tested it with 323 adults living in Kenya. People rated how safe they felt across different settings and life stages, using a color bar going from red (unsafe) to green (safe). The scale is reliable and captures how safe people feel across childhood and adulthood. Feeling safer is associated with lower anxiety and stress, stronger personal resilience, and better memory. Women and adults in poverty report feeling less safe. These patterns were observed in a single study but suggest that cumulative effects of feeling safe can be measured and connected to brain health. Feeling safe seems to impact healthy brain aging, so should be further studied.

## Abstract (web summary)

Blackmon et al. develop and validate the Lifetime Sense of Safety (LSS) scale, a brief visual analog measure of subjective safety across childhood and adulthood, in 323 Kenyan adults. The scale shows good reliability, a two-factor structure, and associations with stress, resilience, and memory.

## Introduction

A sense of safety is an internal experience of feeling secure, protected, and free from harm^1^. It encapsulates feelings of protection from physical and psychosocial threats, whether real, imminent, imagined, or anticipated^2^. Rather than merely the absence of threat, safety is an active biological process, supported by prefrontal and limbic networks in concert with the parasympathetic nervous system^3,4^. Feeling safe facilitates the type of human risk-taking that is critical for social bonding, affect regulation, learning, and cognitive development^5^. Throughout the lifespan, experiences of safety accumulate to shape stress response systems, resilience, and cognitive trajectories in ways that are only beginning to be understood as relevant for late-life brain health.

Brain health refers to the preservation of cognitive functioning and psychological well-being that enable individuals to realize their full potential and to adapt to stressors across the lifespan, consistent with the World Health Organization’s global action plan on dementia prevention^6^. Operationalized in this way, brain health is a multidimensional construct that spans: (1) psychological wellbeing, as commonly indexed by depression and anxiety scales; (2) allostatic load, as commonly indexed by perceived stress and stressful life events; (3) protective capacity, as commonly indexed by measures of psychological resilience; and (4) cognitive function, as commonly indexed by objective memory performance.

The brain is the primary organ that tracks and responds to environmental signaling of safety and threat. Distinct neural circuits process safety versus fear signals^7^. The ventromedial prefrontal cortex (vmPFC) is critical for the formation of safety representations^8^. In contrast, fear responses engage amygdala-centered networks that, when chronically activated, can damage hippocampal neurons and accelerate neurodegeneration^9^. There is emerging evidence that safety may provide a protective buffer between chronic stress and neurodegeneration. Measures of objective^10^ and subjective^11^ neighborhood safety have been shown to protect against cognitive decline in older adults, possibly by calibrating the stress response system and promoting neuroplasticity^12^. However, this is grossly understudied due to limited tools for measuring safety.

Tools for measuring safety should be relevant across global populations. Large-scale modeling of the personal, social, and environmental exposome on brain aging requires tools that are feasible across different education levels, languages, and cultures^13^. This is particularly pertinent across the global south, given the rapid rise in aging populations and forecasted rise in neurodegenerative diseases^14^. Although adverse experiences and environmental threats are considered important constructs within the exposome, psychological safety is often overlooked. In Kenya, extensive research exists on specific safety domains, such as patient safety, road safety, and food security, but there is minimal research on safety as a psychological construct affecting mental health and cognitive aging. One study from pan-African settings shows that feeling safe at school and home is associated with higher resilience and lower depression^15^. This supports the relevance of safety as a meaningful construct for exposome research in African contexts, which can inform the development of interventions to bolster resiliency and promote healthy aging^13,16,17^.

The profound influence of early life experiences on later health outcomes suggests that the measurement approach to safety should incorporate chronic lifespan effects^18^. Positive childhood experiences, including feeling safe and protected at home, buffer against adverse childhood experiences and independently predict adult mental health^19^. Importantly, the effects of chronic safety and positive experiences accumulate across developmental stages: childhood safety influences educational attainment and social competence, which builds lifelong cognitive reserve^20^. Yet, existing measures assess only current perceptions of safety^21,22^ in specific environments^23^ or focus solely on trauma^24^, failing to capture the cumulative nature of lifetime safety experiences across different settings that can shape lifespan brain health trajectories.

These critical measurement gaps led to the development of the Lifetime Sense of Safety (LSS) scale, designed to assess safety across nested settings (spanning home and community) and sequential lifespan stages (childhood, adulthood) using a visual analog color-bar response format suitable for diverse literacy levels. The present study has two aims. The first is to examine the psychometric properties of the LSS scale, including its factor structure, internal consistency, and test-retest reliability, in a large sample of Kenyan adults. The second is to evaluate the scale’s associations with established social determinants of health (sex, education level, and multidimensional poverty) and specific indicators of brain health, selected because each is theoretically linked to felt safety, including: (1) anxiety, perceived stress, and negative life events, selected as proximal indicators of threat reactivity and allostatic load^2,5^; (2) depression, selected as a contrasting indicator less tightly linked to threat processing, to probe the specificity of safety-relevant associations; (3) psychological resilience, selected because safety schemas are posited to function as cognitive resources that facilitate adaptive coping; and (4) objective memory performance, selected as a direct measure of brain health. In keeping with the observational, cross-sectional design, these associations are evaluated as correlational rather than causal. In brief, we find that the six-item LSS scale has good psychometric properties in Kenyan adults, that it contains two correlated childhood and adulthood factors, and that higher LSS scores are associated with lower anxiety and stress, higher psychological resilience, and better objective memory, with consistently stronger associations for adulthood than childhood safety.

## Methods

### Study design and setting

This observational cross-sectional study was nested within two larger studies being conducted at Aga Khan University, Brain and Mind Institute in Nairobi, Kenya. The first study (AD-DETECT) aimed to validate a set of questionnaires, scales, cognitive tests, and biofluid biomarkers for Alzheimer’s disease (AD) detection in older Kenyan adults (45+ years). The second study (Brain Resilience Kenya - BRK) aimed to identify biopsychosocial risk factors for accelerated aging in Kenyan adults (35+ years). Both studies involved an early stage of tool adaptation and piloting, which provided an ideal setting for new tool development. All data were collected in private, distraction-free testing rooms within an outpatient clinic setting at Aga Khan University Hospital.

### Ethics approval and consent

Ethical approval was obtained from the Aga Khan University Institutional Scientific and Ethics Review Committee (AD-DETECT: 2023/ISERC-135; BRK: 2023/ISERC-125) and the National Commission for Science, Technology, and Innovation (AD-DETECT: NACOSTI/P/24/33491; BRK License: NACOSTI/P/25/418163). Research was conducted in accordance with the Declaration of Helsinki. Written consent was obtained from all participants.

### Scale development

The LSS was collaboratively developed in English and Kiswahili by an American neuropsychologist (KB), a Kenyan nurse practitioner (AN), and a Kenyan clinical psychologist (AG), and reviewed for face validity by an expert team of Kenyan and Ugandan anthropologists, neuroscientists, and psychologists. Item content was guided by Social Safety Theory, which posits that perceived safety across nested social contexts and accumulated lifespan stages calibrates stress and immune systems and shapes long-term health^2,5^. Accordingly, items were designed to capture perceived safety across proximal (home), institutional (school, work), and community (neighborhood) settings, and to distinguish childhood from adulthood experiences. Respondents are asked to retrospectively evaluate perceived safety across life stages and settings using a color bar (red to green) with a corresponding number scale from 0 (unsafety) to 10 (safety).

After initial review and item refinement, the 6-item scale was piloted in a focus group of 10 Kenyan social scientists, anthropologists, and clinicians to elicit feedback on the comprehensibility and cultural appropriateness of the scale items, the response modality (e.g., color bar/number span), and the scoring instructions. Administration instructions and scoring procedures were clarified, with attention to common cultural scenarios. For example, concerns were raised about how to handle “school” and “work” items if a respondent lacked a history of formal education or employment. The decision was made to prorate these items using their flankers (if no formal education for item #2, use the average from items #1 and #3; if no employment for item #5, use the average from items #4 and #6). If a respondent raised concerns about not spending enough time at work or school, administrators were instructed to explain that the quality mattered more than the quantity of time spent. If they attended more than one school or job, they were encouraged to consider where they spent the most time. If respondents expressed reluctance to verbally respond to some items, they were reminded that they could point to the best color bar indicator. These procedures were manualized and integrated into standard operating procedures for the AD-DETECT and BRK studies (Supplementary Note 1).

### Participants

Inclusion and exclusion criteria for community-dwelling medically healthy controls were similar across both studies, with the exception of a lower age limit of 35 years in the BRK study and 45 years in the AD-DETECT study. Participants were purposively recruited to reflect the demographic and socioeconomic composition of Nairobi County by engaging community health promoters in different sub-counties, as well as word-of-mouth and social media advertisements. All participants reported fluency in either Swahili or English, the two official languages of Kenya. Participants were excluded if they self-endorsed psychiatric (mood disorder, schizophrenia, substance use disorder), neurologic (all-cause dementia, Huntington’s disease, Parkinson’s disease, brain cancer, stroke, significant head trauma, epilepsy, multiple sclerosis, delirium), or medical (current systemic illness precluding compliance with study procedures, abnormal laboratory findings) conditions during phone-based pre-screening or office-based re-screening. Participants were also excluded if they scored below a standard cut-off of 10 on the Identification of Dementia in Elderly Africans (IDEA) cognitive screen, which is a low-literacy screening measure with good diagnostic accuracy for dementia in Sub-Saharan Africa^25^.

### Measures

A structured questionnaire set was used to gather information about participant demographics and social determinants of health, including age (using birth date recorded from the National ID card), sex, rural versus urban upbringing, and education level (ISCED 2012). For in-depth characterization of socioeconomic status, we applied standardized indicators and an algorithmic framework derived from the Multidimensional Poverty Index (MPI)^26^ and aligned with indicators previously used in Kenya^27^. Within the MPI framework, the Headcount metric facilitated the categorical designation of multidimensional poverty, using an established cut-off > 0.33^28^.

Participants completed the Patient Health Questionnaire-9 (PHQ-9) as a self-report measure of state depression severity and the Generalized Anxiety Disorder-7 (GAD-7) as a self-report measure of state anxiety severity. These measures are widely used globally and have been translated into Swahili and adapted for use in Kenya^29^. The Connor-Davidson Resilience Scale-10 (CDRS-10) has been validated for use as a measure of personal resiliency in South Africa^30^; therefore, we translated the same 10-item version into Swahili and adapted it for use in Kenya. In addition, we translated the 4-item Perceived Stress Scale (PSS-4)^31^ as a measure of current stress state (past month) and the Life Events List (LEL) as a measure of actual experienced stressors over the past year^32^. We made minimal changes to the wording of these two scales based on focus group feedback collected during the initial BRK study tool adaptation stage.

We objectively assessed memory with the 10-item CERAD word list learning test (total learning, delayed recall)^33^ and the Brave Man story memory test (immediate and delayed recall), which has been translated into Swahili and validated for use in older Kenyan adults^34^. Raw scores for each subtest were converted to z-scores using the sample mean and standard deviation, and the four z-scores were then averaged to yield the composite index. This centering-and-averaging approach is standard for creating composite cognitive indices in aging research^35^.

### Statistics and reproducibility

Analyses used the full analytic sample of *N* = 323 adults who completed the LSS scale. Replicates correspond to individual participants, with no technical replicates. The psychometrics of LSS scale items were evaluated for missingness, outliers, internal consistency (Cronbach’s alpha and McDonald’s omega), and factor structure (exploratory factor analysis). Test-retest reliability was computed with Pearson correlation in a subset of *N* = 58 participants who completed the LSS scale again following a 12-month interval. The exploratory factor analysis (EFA) used principal axis factoring (PAF) as the extraction method. We chose EFA rather than confirmatory factor analysis (CFA) because the LSS is a new scale with no prior psychometric data and no previously validated factor structure. Factor retention was guided by parallel analysis and the scree plot criterion. Oblimin (oblique) rotation was used because the two factors were expected to be correlated, given the developmental continuity between childhood and adulthood safety. Criteria for adequate factor loadings were set at loading ≥ 0.40. Sampling adequacy was assessed with the Kaiser-Meyer-Olkin (KMO) measure, with values ≥ 0.80 interpreted as “meritorious”, and factorability with Bartlett’s test of sphericity. To characterize sociodemographic effects, we examined correlations between LSS scores, age, and education, as well as group differences in LSS scores based on sex and multidimensional poverty. To determine whether the LSS scale is positively associated with personal resiliency, we evaluated correlations with the CDRS-10. To assess the protective potential of LSS, we investigated inverse correlations with anxiety (GAD-7), depression (PHQ-9), negative life events, and perceived stress (PSS-4). To evaluate whether LSS is associated with cognitive decline, we assessed the correlation between LSS scores and the composite memory index. Correlations with self-report and objective performance measures were performed with and without adjustment for age, sex, and education, given known associations between these demographic factors and mental health and cognition. Unadjusted data are reported in their original scale metrics; adjusted data are reported as standardized residuals. No adjustment for multiple comparisons was applied, consistent with the scale validation aims. All analyses were performed with SPSS Version 30, with a statistical threshold of *p* < 0.05, and are reproducible from the anonymized de-identified dataset available from the corresponding author upon reasonable request (see Data Availability statement).

## Results

### Sample characteristics

Cognitive screening of controls resulted in 3/95 (3.1%) exclusions from AD-DETECT and 11/245 (4.4%) exclusions from BRK. An additional 3/326 ( < 1%) opted not to complete the LSS scale. The resulting sample of *N* = 323 adults ranged in age from 35 to 84 years (mean = 52.58; sd=11.58) and included 178 females (55.1%), 144 males (44.6%), and one person who self-identified as intersex (0.3%). Most participants were raised in an urban (*N* = 281) versus rural (*N* = 41) setting. The distribution in sex, education level, and multidimensional poverty is generally commensurate with the 2019 Census data from Nairobi County within a 15% margin; except for a lower proportion of adults with a primary school education and a higher proportion of adults with a college education or above (Table 1).

**Table 1:** Sample Characteristics

Feature	Sample N, (%)	% 2019 Census, Nairobi County	Proportionate to Census ( ± 15%)
Sex			
Female	178 (55.1%)	50.14%	Commensurate
Male	144 (44.6%)	49.86%	Commensurate
Intersex	1 (0.3%)		
Education Level			
Pre-primary	5 (1.5%)	3.03%	Commensurate
Primary	58 (18.0%)	33.25%	Below
Secondary	96 (29.7%)	33.93%	Commensurate
Technical / Vocational	77 (23.8%)	13.20%	Commensurate
University Degree and Above	87 (26.9%)	8.83%	Above
Multidimensional Poverty			
Headcount (Incidence)	82 (25.3%)	25.4%	Commensurate

Sample demographics are compared with the 2019 Census data from Nairobi County and considered commensurate if within a 15% margin.

### Missingness and interpolation

Only 6/328 (1.8%) people did not complete items #2 (*N* = 4) or #5 (*N* = 2). Missing data were interpolated with the average from each flanking item, as specified in the manualized scoring instruction.

### Psychometrics

**Fig. 1:**
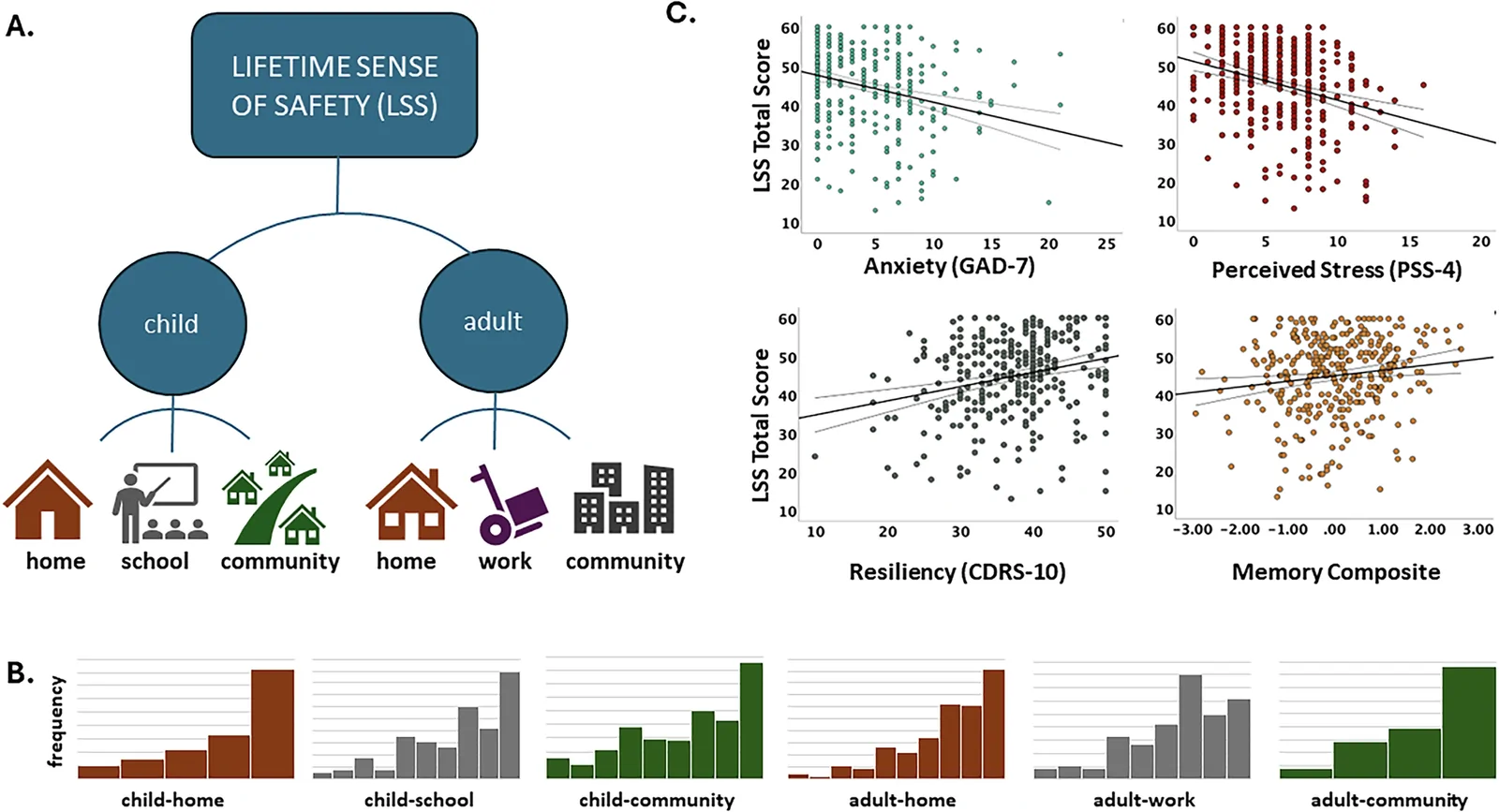
Psychometric structure and correlates of the Lifetime Sense of Safety (LSS) scale. **A** In *n* = 323 adults, the 6-item LSS scale has a two-factor structure, with strong positive loadings of items 1–3 on a “childhood safety” factor and items 4-6 on an “adulthood safety” factor. **B** Total LSS scores are negatively correlated with current anxiety (*n* = 323) and perceived stress (*n* = 323) and positively correlated with personal resiliency (*n* = 323) and objective memory performance (*n* = 322), after adjustment for age and education.

Skewness and kurtosis values for all items fall within –1.5 and 1.5, with a consistent negative skew (−0.78 to –1.1). Item averages range from 7.28 to 7.68 with a concentration of responses in the upper range of the scale (7-10), reflecting the community-dwelling, medically healthy nature of the sample (see Fig. 1B). The internal consistency of the 6 LSS scale items is good (α = 0.83; ω = 0.82). Total LSS scale scores range from 13 to 60 (mean = 44.94; sd = 10.48). In a subset of *N* = 58 participants who were administered the scale at two different time points 12 months apart, test-retest reliability is acceptable (r = 0.74; *p* = 4.73E-11). Sampling adequacy (KMO = 0.82) and item cross-correlations (Bartlett’s test=734; *p* = 1.19E-146) show suitability for exploratory factor analysis. Oblimin rotation reveals two latent factors, accounting for 72.22% of scale variance (Fig. 1A). Childhood items 1-3 exhibit high loadings on Factor 1 (0.83-0.90). Adulthood items 4-6 exhibit high loadings on Factor 2 (0.74–0.92). Therefore, two additional scale indices were created for childhood sense of safety, which range from 3 to 30 (mean=22.42; sd=6.55), and for adulthood sense of safety, which range from 3 to 30 (mean=22.62; sd=5.38). There is a positive correlation between childhood and adulthood sense of safety (r = 0.51, *p* = 3.98E-23). Full results from exploratory principal axis factoring are provided in Table 2.

**Table 2:** Results from exploratory principal axis factoring with oblimin rotation

Total variance explained						
	Initial eigenvalues			Rotated sum of squared loadings		
Component	Total	% of Variance	Cumulative %	Total	% of Variance	Cumulative %
1	3.27	54.45	54.45	2.79	54.45	54.45
2	1.06	17.77	72.22	2.58	17.77	72.22
3	0.59	11.79	83.54			
4	0.43	8.65	92.18			
5	0.39	7.82	100.00			
Rotated component pattern matrix						
			Principal components			
Item			Component 1		Component 2	
Childhood safety: home			0.90			
Childhood safety: school			0.84			
Childhood safety: community			0.83			
Adult safety: home					0.77	
Adult safety: work					0.92	
Adult safety: community					0.74	

Exploratory factor analysis of the Lifetime Sense of Safety (LSS) scale in *n* = 323 adults showing eigenvalues, variance explained, and rotated component loadings for the two-factor solution. Loadings below 0.40 are suppressed.

### Demographic associations

Total LSS scores are not associated with age (r = 0.02; *p* = 0.70) but are positively associated with education (rho=0.25; *p* = 4.78E‑6). This education correlation is present for childhood sense of safety (rho=0.16, *p* = 0.005) but is stronger for adulthood sense of safety (rho=0.25, *p* = 8.66E‑6). Females report a lower sense of safety than males [t(320) = -2.27; *p* = 0.02]. This association with sex is not present for childhood sense of safety [t(320) = -1.65; *p* = 0.10] but is present for adulthood [t(320) = -2.15; *p* = 0.03] sense of safety. There is no difference in LSS scores between those raised in rural versus urban settings [t(320) = -1.6; *p* = 0.12]. Participants classified as multidimensionally poor report a lower sense of safety than those above the poverty threshold [t(286) = 5.37; p3.75E-7]. The association between multidimensional poverty and reduced sense of safety is present for childhood [t(286) = -3.61; *p* = 4.42E‑4] and adulthood [t(286) = -5.23; *p* = 7.48E-7] sense of safety. Correlation coefficients and group-difference estimates are consistently larger in magnitude for adulthood than for childhood sense of safety.

### Divergent and convergent validity

Total LSS scores are not associated with depression (r = -0.11; *p* = 0.06) but are negatively correlated with anxiety (r = -0.28; *p* = 3.89E-7), perceived stress (r = -0.30, *p* = 3.25E-8), and negative life events over the past year (r = -0.21, *p* = 1.36E‑3). Conversely, total LSS scores are positively correlated with personal resiliency (r = 0.25, *p* = 4.71E‑6) and objective memory performance (r = 0.26; *p* = 2.39E-6) (Fig. 1C). This pattern of convergent associations between safety and markers of anxiety, stress, resiliency, and memory, as well as divergent null association with depression, are present after controlling for variance contributed by age, sex, and education (Table 3).

**Table 3:** Associations between LSS and neuropsychological markers of brain health

Feature	Summary Statistics	LSS Total Score	LSS Childhood	LSS Adulthood
	mean (sd)	r-value (p-value)	r-value (p-value)	r-value (p-value)
Anxiety (GAD-7)	3.87 (4.23)	-0.28*** (*p* = 3.89E-7)	-0.19*** (*p* = 4.60E‑4)	-0.30*** (*p* = 6.57E-8)
Anxiety (GAD-7), adjusted^a^	0.00 (1.0)	-0.24*** (*p* = 1.26E‑5)	-0.17** (*p* = 0.003)	-0.25*** (*p* = 3.64E‑6)
Perceived Stress (PSS-4)	6.18 (3.15)	-0.30*** (*p* = 3.25E-8)	-0.15** (*p* = 0.008)	-0.40*** (*p* = 1.14E-13)
Perceived Stress (PSS-4), adjusted^a^	0.00 (1.0)	-0.24*** (*p* = 1.32E‑5)	-0.10 (p = 0.08)	-0.34*** (p = 5.21E-10)
Negative Life Events (LEL)	3.76 (3.05)	-0.21** (*p* = 1.36E‑3)	-0.06 (*p* = 0.36)	-0.33*** (*p* = 1.75E-7)
Negative Life Events (LEL), adjusted^a^	0.00 (1.0)	-0.17* (*p* = 0.01)	-0.03 (*p* = 0.70)	-0.30*** (*p* = 4.19E‑6)
Depression (PHQ-9)	4.37 (4.45)	−0.11 (*p* = 0.06)	-0.06 (*p* = 0.25)	-0.12* (*p* = 0.03)
Depression (PHQ-9), adjusted^a^	0.00 (1.0)	-0.05 (*p* = 0.35)	-0.03 (*p* = 0.65)	-0.06 (*p* = 0.27)
Resilience (CDRS-10)	37.60 (7.15)	0.25*** (*p* = 4.71E‑6)	0.20*** (*p* = 3.76E‑4)	0.25*** (*p* = 1.41E‑5)
Resilience (CDRS-10), adjusted^a^	0.00 (1.0)	0.24*** (*p* = 1.02E‑5)	0.19*** (*p* = 5.87E‑4)	0.23*** (*p* = 2.54E‑5)
Memory Index, standardized	0.00 (0.81)	0.26*** (*p* = 2.39E 6)	0.19*** (*p* = 5.24E‑4)	0.25*** (*p* = 8.13E‑6)
Memory Index, standardized, adjusted^a^	0.00 (1.00)	0.15** (*p* = 0.009)	0.11* (*p* = 0.04)	0.15** (*p* = 0.009)

^*^p < 0.05; **p < 0.001; ***p < 0.001

^a^Standardized residuals after regressing out variance associated with age, sex, and education.

Generalized Anxiety Disorder-7 items (GAD-7), *n* = 323 adults; Perceived Stress Scale-4 items (PSS-4), *n* = 323 adults; Life Events List (LEL), *n* = 233 adults; Patient Health Questionnaire – 9 (PHQ-9), *n* = 320 adults; Connor-Davidson Resilience Scale-10 (CDRS-10), *n* = 323 adults; Memory Index, *n* = 322 adults.

Pearson correlations (two-sided) were used to evaluate associations between LSS and neuropsychological markers of brain health. No adjustments were made for multiple comparisons.

A similar pattern is observed when childhood and adulthood senses of safety are separately considered. A higher sense of childhood safety at home, school, and in the surrounding neighborhood is associated with lower levels of adult anxiety (r = -0.19; *p* = 4.60E‑4) and perceived stress (r = -0.15, *p* = 0.008), as well as a higher degree of personal resiliency (r = 0.20; *p* = 3.76E‑4). There is no association between childhood sense of safety and adulthood depression or negative life events. Childhood sense of safety is positively correlated with objective memory performance in adulthood (r = 0.19; *p* = 5.24E‑4). This pattern of associations is retained after controlling for age, sex, and education, except perceived stress, which reduced to non-significance (Table 3). For adulthood, a higher sense of safety at home, workplace, and neighborhood is associated with lower anxiety (r = −0.30, *p* = 6.57E-8), perceived stress (r = −0.40; *p* = 1.14E-13), and negative life events (r = −0.33, *p* = 1.75E-7), as well as a higher degree of personal resiliency (r = 0.24; *p* = 1.41E‑5) and objective memory performance (r = 0.25; *p* = 8.13E‑6). This pattern of adulthood associations is retained after controlling for age, sex, and education. Compared to childhood, the strength of all correlations is consistently greater for adulthood's sense of safety (Table 3).

## Discussion

A sense of safety is essential for lifelong learning, enrichment, and well-being. When we feel safe, we can explore, take risks, and learn, which provides an adaptive advantage and potentially contributes to brain resilience. Yet, psychological safety is not typically considered part of the exposome in brain aging research. To address this gap, we developed and validated a measure of accumulated felt safety across the lifespan in cognitively unimpaired Kenyan adults. Our findings demonstrate that the LSS scale is a psychometrically sound instrument, with good internal consistency and test-retest reliability. The emergence of two latent factors, childhood and adulthood safety, aligns with developmental theories suggesting that safety experiences consolidate differently across life stages^18^. This factor structure enables researchers to examine both cumulative lifetime and stage-specific associations with health outcomes. Our findings suggest that while both periods matter, adulthood safety shows stronger associations with current psychological states and memory performance, potentially due to temporal proximity to outcomes and/or the cumulative impact of safety experiences across life stages and settings.

We found that a higher lifetime sense of safety is associated with lower anxiety and stress, as well as higher personal resiliency. This provides initial empirical support for felt safety as a component of the “protectome”, a term we propose to capture aggregate protective factors (education, occupational complexity, leisure activities, social engagement, green/blue spaces) that may buffer against stress and cognitive decline^13,20^. Given its reliance on self-report, the LSS scale likely reflects personal safety schemas, cognitive representations formed through accumulated experiences of stress and influenced by social learning^6^. These schemas develop iteratively, with early experiences shaping internal working models that are continuously updated by new safety experiences throughout the lifespan. Positive safety schemas may function as cognitive resources that facilitate adaptive responses to current challenges. The two-factor (childhood, adulthood) structure of the LSS scale suggests that safety schemas may be developmentally organized, with early experiences creating foundational templates that are then modified but not replaced by adult experiences. This aligns with attachment theory’s proposition that internal working models established in childhood continue to influence but do not fully determine adult relationship patterns and stress responses^36–38^.

Notably, we found an association between LSS and objective memory performance. This association is present for both childhood and adulthood sense of safety, which raises the possibility that early safety experiences establish neural foundations for lifelong cognitive reserve, alongside other established reserve indicators such as nutrition^39^ and education^40^. Although the cross-sectional nature of our study design precludes causal or directional interpretation, our findings are consistent with results from a 5-year longitudinal study of safety and cognitive trajectories in older adults that found subjective indicators of neighborhood safety are predictive of cognitive stability (i.e., less cognitive decline)^11^. Interestingly, subjective safety indicators were more predictive than objective indicators^11^. This suggests that subjective safety measures can provide complementary information that reflects how individuals process and cope with their environments based on internal safety schemas, available social support, and personal resilience.

The positive association between safety and memory can be understood by considering how safety calibrates the stress system to respond efficiently to threat and attenuate lifespan allostatic load. There is a large body of mechanistic work in animals and humans that links safety experiences to neuroplasticity^41–44^, in parallel with animal and human studies that link chronic stress exposure to dysregulated stress and immune system responsiveness^45–53^. Prefrontal regions provide a critical substrate for safety representations^3^. The vmPFC actively integrates threat and protective information to meta-represent safety schemas, with anterior regions serving as a hub for self-relevant protective information^41^. Connectivity between vmPFC and amygdala-hippocampal-hypothalamic pathways restores or maintains parasympathetic tone during safety conditions^3^. In rodent models, learned safety promotes neuroplasticity through increased hippocampal neurogenesis and brain-derived neurotrophic factor^42^. Learned safety also elicits behavioral anxiolytic effects, such as increased adventurous exploration of an open field^7^. Safety signal learning is replicated in humans as a means to reduce fear-related behaviors and encourage exploration, even in the presence of threat^43,44^.

Conversely, a chronic sense of unsafety contributes to abnormal stress and immune system responsiveness, with an impact on brain health and cognitive functioning^54–56^. Chronic stressor exposure is associated with abnormal hypothalamic-pituitary-adrenal (HPA) axis activity^45^, decreased glucocorticoid receptor sensitivity^46^, and mitochondrial dysfunction^47^, particularly when stressors occur in early childhood^48^. Adverse early life experiences can reprogram the hippocampal transcriptome and contribute to lasting effects on memory^49^. In addition, a higher degree of adverse childhood experiences is linked to an exaggerated systemic and intracellular inflammatory response to repeated psychosocial stress in adulthood^50^, with findings of elevated plasma interleukin-6^51^ and cortisol^52^ reactivity to acute stressors. Critical windows for stress programming may not be limited to early childhood. When middle-aged rats are administered high levels of glucocorticoids for extended periods, they show resulting memory deficits^53^. These human and animal findings are consistent with the pattern of associations we observed but linking human self-reported safety experiences to stress system responding will require studies that pair the LSS scale with neural and physiological measures in the same individuals.

Interestingly, we did not find a strong association between LSS and depression, despite negative correlations with anxiety and stress. This provides preliminary evidence for divergent validity in a single sample, suggesting that safety may specifically associate with stress regulation rather than mood regulation per se. The safety construct, as operationalized by the LSS scale, may be more closely related to threat-detection and threat-regulation systems, while depressive symptoms are shaped by a broader set of losses and biological vulnerabilities that the LSS scale was not designed to capture. However, it is also possible that the medically and psychiatrically healthy nature of our adult sample may have truncated the upper end of depressive severity and limited statistical power to detect an association.

The observed associations between LSS scores and sociodemographic factors illuminate how structural inequalities shape safety experiences. Findings of lower sense of safety in women and those experiencing multidimensional poverty are consistent with the known relationships between psychological safety, financial resources, and structural vulnerability^18^. Safety is influenced by one’s position within social hierarchies that determine access to resources, security, and agency^2^. Being a woman and being poor can mean being at the mercy of others for financial security and navigating systems that may not provide adequate protection. Stronger correlations between social determinants of health and felt safety in adulthood suggest that structural inequalities may accumulate or intensify over the life course, particularly as individuals assume adult responsibilities without adequate resources or social protection.

The positive association with education, particularly for adulthood safety, likely reflects both the protective role of education itself, through enhanced economic opportunities and social capital, as well as serving as a proxy for childhood environments that were safe enough to enable educational attainment. Thus, perceived safety could be considered as both an outcome of social position and a mechanism through which social determinants influence health across generations. These results provide preliminary support for the use of the LSS scale to capture aspects of the lifespan “protectome” that may serve as a buffer against stress, trauma, and age-related cognitive decline. Although the two-factor scale structure requires further confirmatory factor analyses in independent samples, the childhood and adulthood index scores could potentially be used independently, depending on the research question. For studies of childhood adversity and developmental trauma, the childhood sense of safety score provides a strength-based complement to deficit-focused measures like the Adverse Childhood Experiences questionnaire^24^. For research on current environmental influences on health, the adulthood sense of safety score captures contemporary experiences across multiple settings, including the workplace. When aging is the outcome measure, we recommend using the total score as it considers the lifespan accumulated sense of safety and shows the strongest relationship with stress, resiliency, and cognitive outcome measures.

The visual analog scale using a color gradient from red (unsafe) to green (safe) represents a methodological innovation with potential for global adaptation. Color associations with safety and danger show remarkable cross-cultural consistency^57–59^. The intuitive nature of the color bar may reduce literacy barriers and social desirability bias compared to verbal response scales, making it particularly suitable for use in diverse populations with varying educational backgrounds. Successful validation in Kenya, where participants ranged from no formal education to postgraduate degrees, suggests potential applicability across other low- and middle-income country settings where traditional psychometric tools may be less accessible or culturally appropriate.

Several limitations warrant consideration and next-step guidance for future work. First, the cross-sectional design precludes causal inferences about the relationship between lifetime sense of safety and health outcomes. Second, retrospective assessment of childhood safety may be subject to recall and mood biases, though moderate test-retest reliability suggests reasonable stability of subjective safety appraisals, and the null association with depression argues against mood-congruent recall bias. Third, shared method variance from self-report can inflate correlations among psychological scales; however, the observed association between LSS scores and objective memory performance mitigates this concern. Fourth, the two-factor solution was derived from exploratory factor analysis in a single sample and should be considered provisional pending confirmatory analysis in independent samples. Finally, generalizability is constrained by sampling: participants were recruited from an urban setting; adults with primary school education only were underrepresented; and the protocol was limited to Swahili and English speakers. Exclusion of people with cognitive impairment and psychiatric disorders may have truncated the range of responses across measures but also allows for estimation of reliability, factor structure, and associations with social determinants of health without the confounding state-dependent influence of psychiatric or neurocognitive disorders.

Future research is needed to address these limitations. First, confirmatory factor analysis and tests of measurement variance across sex, socioeconomic, and clinical groups are needed before between-group comparisons can be interpreted with confidence. Second, translation, adaptation, and validation across different cultural, linguistic, and rural settings will determine whether the two-factor solution and observed associations replicate beyond urban Kenyan adults. Third, longitudinal follow-up is needed to determine whether baseline LSS scores prospectively predict changes in cognition, mental health, and biological markers of aging, thereby clarifying whether a lifetime sense of safety confers benefits on healthy brain aging. Finally, integrating the LSS scale with biomarkers of accelerated aging, including inflammatory markers, cortisol dynamics, and neuroimaging indices of brain health, ould shed light on biological pathways linking lifetime safety to brain health outcomes.

## Conclusion

The LSS scale offers a promising approach to measuring subjective safety across the lifespan for brain aging research. In this initial validation sample of cognitively unimpaired adults in Nairobi, the scale shows good internal consistency, acceptable test-retest reliability, and a coherent two-factor structure distinguishing childhood and adulthood safety, with total scores associating in expected directions with anxiety, perceived stress, personal resilience, and objective memory performance. Its brief, visually accessible format appears feasible across diverse educational backgrounds. Pending further validation across various languages, cultures, and clinical groups, it is potentially well-suited for inclusion in epidemiological studies of the exposome alongside objective safety indicators and biological markers of stress, inflammation, and neurodegeneration. This promising first-step validation illustrates the LSS scale’s potential to advance research on how safety relates to resilience and cognitive aging across diverse global populations.

## Supplementary information

Transparent Peer Review file

Supplementary Information

## Data Availability

The dataset contains potentially sensitive information from a study conducted in Kenya; therefore, access to de-identified participant-level records will be subject to a data-sharing agreement and approval by the Aga Khan University Nairobi Institutional Scientific and Ethics Review Committee, in accordance with Kenyan data-protection law. Requests for access to the underlying dataset can be directed to the corresponding author and will be acknowledged within two weeks of receipt, with a decision communicated within eight weeks, subject to the meeting schedule of the Institutional Scientific and Ethics Review Committee. Under the data-sharing agreement, approved recipients must use the data solely for the agreed research purpose; must not attempt to re-identify participants; must not redistribute or further disclose the data, and must ensure that any authorized personnel are bound by equivalent obligations; must apply appropriate safeguards to protect the data; and must return or destroy the data at the end of the agreed period. The full LSS instrument (English and Swahili versions), and the scoring key, are provided as Supplementary Note 1 in the Supplementary Information; it is available free of charge for research use, with a request that users cite this paper in any resulting publications or presentations.

## References

[1] Pessi, A. B. et al. From safety to sense of safety. *Front. Psychol.* **16**, 1624676. 10.3389/fpsyg.2025.1624676 (2025). PMC12391010 40893846

[2] Slavich, G. M. Social safety theory: a biologically based evolutionary perspective on life stress, health, and behavior. *Annu. Rev. Clin. Psychol.* **16**, 265–295 (2020). 10.1146/annurev-clinpsy-032816-045159 PMC7213777 32141764

[3] Kong, E., Monje, F. J., Hirsch, J. & Pollak, D. D. Learning not to fear: neural correlates of learned safety. *Neuropsychopharmacology* **39**, 515–527 (2014). 10.1038/npp.2013.191 PMC3895233 23963118

[4] Harrison, et al. Human ventromedial prefrontal cortex and the positive affective processing of safety signals. *Neuroimage* **152**, 12–18 (2017). 10.1016/j.neuroimage.2017.02.080 28254509

[5] Slavich, G. M. et al. Social safety theory: conceptual foundation, underlying mechanisms, and future directions. *Health Psychol. Rev.* **17**, 5–59 (2023). 10.1080/17437199.2023.2171900 PMC10161928 36718584

[6] Seeher, K. M. The WHO’s Global action plan on Dementia. *Alzheimer's Dement.* **21**, e110731 (2025).

[7] Rogan, M. T., Leon, K. S., Perez, D. L. & Kandel, E. R. Distinct neural signatures for safety and danger in the amygdala and striatum of the mouse. *Neuron* **46**, 309–320 (2005). 10.1016/j.neuron.2005.02.017 15848808

[8] Bukalo, O. et al. Prefrontal inputs to the amygdala instruct fear extinction memory formation. *Sci. Adv.* **1**, e1500251 (2015). 10.1126/sciadv.1500251 PMC4618669 26504902

[9] Lucassen, P. J. et al. Neuropathology of stress. *Acta Neuropathol.* **127**, 109–135 (2014). 10.1007/s00401-013-1223-5 PMC3889685 24318124

[10] Vassilaki, M. et al. Association of neighborhood socioeconomic disadvantage and cognitive impairment. *Alzheimer's Dement.* **19**, 761–770 (2023). 10.1002/alz.12702 PMC9722980 35666244

[11] Hyun, J. et al. Perceived but not objective measures of neighborhood safety and food environments are associated with longitudinal changes in processing speed among urban older adults. *BMC Geriatr.* **24**, 551 (2024). 10.1186/s12877-024-05068-0 PMC11197239 38918697

[12] Davidson, R. J. & McEwen, B. S. Social influences on neuroplasticity: stress and interventions to promote well‑being. *Nat. Neurosci.* **15**, 689–695 (2012). 10.1038/nn.3093 PMC3491815 22534579

[13] Udeh-Momoh, C. T. et al. Resilience and brain health in global populations. *Nat. Med.* **31**, 2518–2531 (2025). 10.1038/s41591-025-03846-w PMC13189249 40731089

[14] Livingston, G. et al. Dementia prevention, intervention, and care: 2020 report of the Lancet Commission. *Lancet* **396**, 413–446 (2020). 10.1016/S0140-6736(20)30367-6 PMC7392084 32738937

[15] Bandeira, M., Graham, M. A. & Ebersöhn, L. The significance of feeling safe for resilience of adolescents in sub‑Saharan Africa. *Front. Psychol.* **14**, 1183748 (2023). 10.3389/fpsyg.2023.1183748 PMC10469746 37663363

[16] World Health Organization. Global action plan on the public health response to dementia 2017–2025. (WHO, 2017).

[17] Udeh-Momoh, C. T. et al. Africa-FINGERS Study Team. Dementia risk reduction in the African context: multi-national implementation of multimodal strategies to promote healthy brain aging in Africa (the Africa-FINGERS project). *Alzheimer's Dement.* **20**, 8987–9003 (2024). 10.1002/alz.14344 PMC11667543 39511921

[18] Nelson, C. A. & Gabard‑Durnam, L. J. Early adversity and critical periods: neurodevelopmental consequences of violating the expectable environment. *Trends Neurosci.* **43**, 133–143 (2020). 10.1016/j.tins.2020.01.002 PMC8092448 32101708

[19] Bethell, C. et al. Positive childhood experiences and adult mental and relational health in a statewide sample. *JAMA Pediatr.* **173**, e193007 (2019). 10.1001/jamapediatrics.2019.3007 PMC6735495 31498386

[20] Stern, Y. et al. Defining and investigating cognitive reserve, brain reserve, and brain maintenance. *Alzheimer's Dement.* **16**, 1305–1311 (2020). 10.1016/j.jalz.2018.07.219 PMC6417987 30222945

[21] Spinoni, M., Zagaria, A., Pecchinenda, A. & Grano, C. Factor structure, construct validity, and measurement invariance of the Neuroception of Psychological Safety Scale (NPSS). *Eur. J. Investig. Health Psychol. Educ.* **14**, 2702–2715 (2024). 10.3390/ejihpe14100178 PMC11507099 39452173

[22] Dabbous, M. et al. Psychometric validation and correlates of the personal safety perception scale (PSPS‑26) as a multidimensional measure of perceived safety. *Sci. Rep.* **15**, 28376 (2015). 10.1038/s41598-025-13050-y PMC12321989 40760070

[23] Dabaghi, S., Zandi, M., Ebadi, A., Abbaszadeh, A., & Rohani, C. Development and psychometric evaluation of the safety feeling scale in adult patients at hospital: exploratory sequential mixed method. *Nurs. Open.* **10**, 6165–6174 (2023). 10.1002/nop2.1850 PMC10416024 37246347

[24] Felitti, V. J. et al. Relationship of childhood abuse and household dysfunction to many of the leading causes of death in adults: the Adverse Childhood Experiences (ACE) Study. *Am. J. Prev. Med.* **14**, 245–258 (1998). 10.1016/s0749-3797(98)00017-8 9635069

[25] Gray, W. K. et al. Population normative data for three cognitive screening tools for older adults in sub‑Saharan Africa. *Dement. Neuropsychol.* **15**, 339–349 (2021). 10.1590/1980-57642021dn15-030005 PMC8485647 34630921

[26] Alkire, S. & Foster, J. Counting and multidimensional poverty measurement. *J. Public Econ.* **95**, 476–487 (2011).

[27] Oxford Poverty and Human Development Initiative. Kenya country briefing. Oxford: OPHI (University of Oxford, 2024).

[28] Alkire, S., Kanagaratnam, U. & Suppa, N. A methodological note on the Global Multidimensional Poverty Index (MPI) 2024 changes over time results for 86 countries. OPHI MPI Methodol Note 60. (Oxford Poverty and Human Development Initiative, 2024).

[29] Odero, S. A. et al. A. Psychometric evaluation of PHQ‑9 and GAD‑7 among community health volunteers and nurses/midwives in Kenya following a nation‑wide telephonic survey. *Front. Psychiatry* **14**, 1123839 (2023). 37324823 10.3389/fpsyt.2023.1123839 PMC10264862

[30] Pretorius, T. B. & Padmanabhanunni, A. Validation of the Connor-Davidson Resilience Scale-10 in South Africa: item response theory and classical test theory. *Psychol. Res. Behav. Manag.* **15**, 1235–1245 (2022). 35603351 10.2147/PRBM.S365112 PMC9122044

[31] Ezzati, A. et al. Validation of the perceived stress scale in a community sample of older adults. *Int. J. Geriatr. Psychiatry* **29**, 645–652 (2014). 10.1002/gps.4049 PMC4013212 24302253

[32] Cohen, S., Tyrrell, D. A., & Smith, A. P. Psychological stress and susceptibility to the common cold. *N. Engl. J. Med.* **325**, 606–612 (1991). 10.1056/NEJM199108293250903 1713648

[33] Fillenbaum, G. G. & Mohs, R. CERAD (Consortium to Establish a Registry for Alzheimer’s Disease) neuropsychology assessment battery: 35 years and counting. *J. Alzheimer's Dis.* **93**, 1–27 (2023). 10.3233/JAD-230026 PMC10175144 36938738

[34] Riang’a, R. M. et al. Contextualization of Harmonized Cognitive Assessment Protocol (HCAP) in an aging population in rural low‑resource settings in Africa: experiences and strategies adopted to optimize effective adaption of cognitive tests in Kenya. *Alzheimer's Dement.* **21**, e70552 (2025). 40835849 10.1002/alz.70552 PMC12367447

[35] Stricker, N. H. et al. Mayo-PACC: a parsimonious preclinical Alzheimer’s disease cognitive composite comprised of public-domain measures to facilitate clinical translation. *Alzheimer's Dement.* **19**, 2575–2584 (2023). 10.1002/alz.12895 PMC10272034 36565459

[36] Bowlby, J. *Attachment and Loss* (Basic Books, 1969).

[37] Fraley, R. C. Attachment stability from infancy to adulthood: meta‑analyses and dynamic modeling of developmental mechanisms. *Pers. Soc. Psychol. Rev.* **6**, 123–151 (2002).

[38] Widom, C. S., Czaja, S. J., Kozakowski, S. S. & Chauhan, P. Does adult attachment style mediate the relationship between childhood maltreatment and mental and physical health outcomes? *Child Abuse Negl.* **76**, 533–545 (2018). 10.1016/j.chiabu.2017.05.002 PMC5685930 28522128

[39] Stein, et al. Nutritional supplementation in early childhood, schooling, and intellectual functioning in adulthood: a prospective study in Guatemala. *Arch. Pediatr. Adolesc. Med.* **162**, 612–618 (2018). 10.1001/archpedi.162.7.612 PMC3733080 18606931

[40] Fjell, et al. Reevaluating the role of education on cognitive decline and brain aging in longitudinal cohorts across 33 Western countries. *Nat. Med.* **31**, 2967–2976 (2025). 10.1038/s41591-025-03828-y PMC12888805 40721513

[41] Tashjian, S. M., Cussen, J., Deng, W., Zhang, B. & Mobbs, D. Subregions in the ventromedial prefrontal cortex integrate threat and protective information to meta-represent safety. *PLoS Biol.* **23**, e3002986 (2025). 10.1371/journal.pbio.3002986 PMC11730396 39804855

[42] Pollak, D. D. et al. An animal model of a behavioral intervention for depression. *Neuron* **60**, 149–161 (2008). 18940595 10.1016/j.neuron.2008.07.041 PMC3417703

[43] Jovanovic, T. et al. Fear potentiation and fear inhibition in a human fear‑potentiated startle paradigm. *Biol. Psychiatry* **57**, 1559–1564 (2005). 15953493 10.1016/j.biopsych.2005.02.025

[44] Meyer, H. C. et al. Ventral hippocampus interacts with prelimbic cortex during inhibition of threat response via learned safety in both mice and humans. *Proc. Natl. Acad. Sci. USA* **116**, 26970–26979 (2019). 31822612 10.1073/pnas.1910481116 PMC6936350

[45] Danese, A. & Lewis, S. J. Psychoneuroimmunology of early‑life stress: the hidden wounds of childhood trauma? *Neuropsychopharmacology* **42**, 99–114 (2017). 27629365 10.1038/npp.2016.198 PMC5143500

[46] Cohen, S. et al. Chronic stress, glucocorticoid receptor resistance, inflammation, and disease risk. *Proc. Natl. Acad. Sci. USA* **109**, 5995–5999 (2012). 22474371 10.1073/pnas.1118355109 PMC3341031

[47] Duchowny, et al. Childhood adverse life events and skeletal muscle mitochondrial function. *Sci. Adv.* **10**, eadj6411 (2024). 10.1126/sciadv.adj6411 PMC10917337 38446898

[48] Humphreys, K. L. et al. Evidence for a sensitive period in the effects of early life stress on hippocampal volume. *Dev. Sci.* **22**, e12775 (2019). 10.1111/desc.12775 PMC6469988 30471167

[49] Baram, T. Z. & Birnie, M. T. Enduring memory consequences of early‑life stress/adversity: structural, synaptic, molecular and epigenetic mechanisms. *Neurobiol. Stress* **33**, 100669 (2024). 10.1016/j.ynstr.2024.100669 PMC11415888 39309367

[50] Schreier, H. et al. Childhood physical neglect is associated with exaggerated systemic and intracellular inflammatory responses to repeated psychosocial stress in adulthood. *Front. Psychiatry* **11**, 504 (2020). 32581878 10.3389/fpsyt.2020.00504 PMC7290130

[51] Carpenter, L. L. et al. Association between plasma IL‑6 response to acute stress and early‑life adversity in healthy adults. *Neuropsychopharmacology* **35**, 2617–2623 (2010). 20881945 10.1038/npp.2010.159 PMC2978751

[52] Carpenter, L. L. et al. Effect of childhood emotional abuse and age on cortisol responsivity in adulthood. *Biol. Psychiatry* **66**, 69–75 (2009). 19375070 10.1016/j.biopsych.2009.02.030 PMC2696583

[53] de Souza‑Talarico, J. N., Marin, M. F., Sindi, S. & Lupien, S. J. Effects of stress hormones on the brain and cognition: evidence from normal to pathological aging. *Dement. Neuropsychol.* **5**, 8–16 (2011). 10.1590/S1980-57642011DN05010003 PMC5619133 29213714

[54] Fulop, T. et al. Immunology of aging: the birth of inflammaging. *Clin. Rev. Allergy Immunol.* **64**, 109–122 (2023). 10.1007/s12016-021-08899-6 PMC8449217 34536213

[55] Oitzl, M. S., Champagne, D. L., van der Veen, R. & de Kloet, E. R. Brain development under stress: hypotheses of glucocorticoid actions revisited. *Neurosci. Biobehav. Rev.* **34**, 853–866 (2010). 10.1016/j.neubiorev.2009.07.006 19631685

[56] Saez‑Sanz, N., Peralta‑Ramirez, I., Gonzalez‑Perez, R., Vazquez‑Justo, E. & Caracuel, A. Resilience, stress, and cortisol predict cognitive performance in older adults. *Healthcare* **11**, 1072 (2023). 10.3390/healthcare11081072 PMC10137485 37107906

[57] Hupka, R. T., Zaleski, Z., Otto, J., Reidl, L. & Tarabrina, N. The colors of anger, envy, fear, and jealousy: a cross‑cultural study. *J. Cross Cult. Psychol.* **28**, 156–171 (1997).

[58] Wogalter, M. S., Conzola, V. C. & Smith‑Jackson, T. Research‑based guidelines for warning design and evaluation. *Appl. Erg.* **33**, 219–230 (2002). 10.1016/s0003-6870(02)00009-1 12164506

[59] Palmer, S. E. & Schloss, K. B. An ecological valence theory of human color preference. *Proc. Natl. Acad. Sci. USA* **107**, 8877–8882 (2010). 20421475 10.1073/pnas.0906172107 PMC2889342

