# Supplementary material for: Development and validation of the lifetime sense of safety scale in Kenyan adults
Source: Commun Med (Lond). 2026 Jun 6;6:483. doi: 10.1038/s43856-026-01715-2 (PMC13575095; doi:10.1038/s43856-026-01715-2)
Supplement: Supplementary file 1 [file 43856_2026_1715_MOESM1_ESM.pdf]

nature portfolio

Peer Review File

Development and Validation of the Lifetime Sense of Safety Scale in Kenyan Adults

Corresponding Author: Dr Karen Blackmon

This file contains all reviewer reports in order by version, followed by all author rebuttals in order by version.

Version 0:

Reviewer comments:

Reviewer #1

(Remarks to the Author)
The paper presents two aims: to evaluate the Lifetime Sense of Safety scale's psychometric properties and to examine associations with established social determinants of brain health in a Kenyan sample. This is an important contribution, as validated measures of safety perception in low- and middle-income contexts are needed to advance research on social determinants of health. My comments are below.

The introduction does not clearly define what constitutes "brain health." Given that this is the central focus of the paper, a clear operational definition is essential.

The study employs a wide range of outcome variables, including the PHQ-9, stress scales, and CERAD word list, yet the extensive introduction does not adequately lay the groundwork for how these multiple outcome indicators map onto the construct of brain health. Further clarification on how these diverse measures collectively capture or relate to brain health would strengthen the conceptual framework of the study. Additionally, summary statistics for all outcome variables would help readers better understand their distributions within the sample.

Regarding the composite memory index, it is unclear which specific subtests were included, whether z-score standardisation was used, or how variability across subtests was handled. Additional details on these procedures would be helpful.

The rationale for adjusting for education requires clarification, particularly given that education also appears to be treated as an outcome in the discussion. This dual role creates conceptual ambiguity that should be addressed.

Please provide detailed information regarding the cutoff criteria for fit indices used in the analysis section. It would be beneficial to include specifics about the methods employed in the Exploratory Factor Analysis (EFA), the statistical tests conducted, and the corresponding cutoff values.

The analysis appears to lack tables that present the factor loadings. Including these would greatly enhance the readability and comprehensiveness of the findings.

A distribution of the responses to the 10-point scale questions would be helpful for understanding the data more thoroughly.

There are concerns regarding the use of Cronbach's alpha. Recent literature suggests that McDonald's omega may provide a more accurate and robust measure of reliability, and the authors should consider this alternative.

The rationale for employing EFA rather than Confirmatory Factor Analysis (CFA) requires further clarification, particularly given that the stated aim is validation. EFA is generally used to develop and explore new scales, whereas CFA is typically employed to validate the factor structure in a new or independent sample. The authors should address why EFA was deemed more appropriate for this study's objectives.

The limitations section does not adequately address the inherent constraints and potential issues associated with using EFA. A discussion of these methodological limitations would strengthen the paper.

The scale was tested in a clinical setting. The authors could discuss the implications for the use of the scale outside of clinical contexts, including its generalisability and applicability to community or population-based settings.

Reviewer #2

(Remarks to the Author)
This manuscript addresses an interesting and potentially important gap in the literature by positioning safety as a social determinant of brain health and by attempting to operationalize this construct in a Kenyan context. The focus is novel, the setting is highly valuable, and the effort to develop a brief, visually accessible measure that may be feasible across education levels is a clear strength. I also appreciated the authors' attempt to link perceived lifetime safety not only to psychological functioning, but also to cognitive outcomes, which gives the paper broader conceptual relevance for global mental health and cognitive aging research. Overall, this manuscript has the potential to make a meaningful contribution. However, there are several major conceptual, methodological, and interpretive issues that should be addressed before the manuscript is suitable for publication.

Overall:
• Your scale relies on self-reported perception of safety in childhood, measured during adulthood, as well as self-reported measures of associated factors. I think this could bias your results, thus should be factored in to the conclusions.
• Throughout the paper, causal language is applied to refer to study aims and results and conclusions. This should be modified to reflect the design of the study.
• Sampling and generalizability are more limited than the framing implies. This is a clinic-based, largely urban Nairobi sample with an overrepresentation of higher education, plus exclusion of anyone self-endorsing common psychiatric conditions. That combination likely truncates both exposure and outcome variability and may inflate or distort associations (for example, safety with anxiety and memory) relative to what you would see in more socioeconomically diverse community samples, rural settings, or among people with current mental health problems. I would strengthen the manuscript by being more explicit that this is an initial validation in a specific sampling frame, and by outlining a concrete next-step validation plan (for example, community sampling, rural counties, inclusion of people with depression or trauma histories, and replication of factor structure and correlates).
• The psychometrics are promising, but key details and sensitivity checks are missing for a scale-validation paper. Right now, the results lean heavily on alpha, EFA, and a single test-retest correlation. I would expect: (a) fuller reporting of the EFA (extraction method, eigenvalues/scree, factor correlation, communalities, cross-loadings), (b) some assessment of measurement performance across education and sex (at minimum, invariance checks or a clear plan to test invariance), and (c) sensitivity analyses around the pro-rating/interpolation rules, since imputing school and work from flanking items may mechanically reinforce the two-factor structure and dampen missingness-related uncertainty. Even a short appendix showing results with and without interpolated cases would help.
Abstract:
• It would be good to have a bit more detail about the theoretical underpinning of this scale. How did you develop it? From where did you derive the items?
• You mentioned you evaluated associations with established social determinants of brain health. If there is space, it would be good to highlight at least some examples of the factors you evaluated for association with safety.
• You mentioned that better memory was associated with psychological safety after adjusting for age and education. Were the other mentioned association not adjusted for these factors? I think the methods should explicitly state the statistical tests conducted for deriving associations.
• You mention effect sizes. It would be good to specify what effects you are referring to. Also, there was no experiment done here, so I think you should use the language appropriate to the test conducted. I am also wondering if you mean that these effects were stronger regarding the association with psychological resilience and cognitive health.
• In your conclusion you mentioned that the scale is short, but nowhere in the abstract is the length of the scale mentioned.
• In your conclusion, you use the word effects, but there was no experiment conducted in your study, so this language is inappropriate.
Introduction:
• You state that the LSS was developed to address measurement gaps which you outline in the previous paragraph. However, according to the current aim, it is unclear how your study addresses these measurement gaps other than the lifespan measurement. For instance, does it focus on aspects of safety other than trauma?
• The second aim of the study which seems to be about the association of your new measure with several psychosocial constructs needs clarification. How is this related to lifetime sense of safety? What is the theoretical and scientific underpinning of the assessment of LSS's association with these factors? How were they selected?

Methods:
• From your description of the scale development, it is unclear how the items were derived and what domains are included. Please elaborate on how was it determined that this scale should be 6 items. Are there 6 domains of safety? Is there any theoretical basis for the selected scale items?
• Your inclusion criteria for languages seems to leave out other language speakers that don't speak Kiswahili or English, i.e. those who might not have gone through the education system, which you mention in paragraph 2 of the scale development section. This could potentially explain the fact that the general population's education level is different from your study's.
• From how the results are currently written, it is not clear why you excluded those with certain health conditions. I imagine this was part of the aims of the larger studies. It seems this would bias your sample towards a significantly higher proportion of healthy people than the general population.
• In the analysis section, you claim that you assessed whether sense of safety confers protection against cognitive decline. However, as your study is observational and cross-sectional, you cannot assess this.

Results:
• In the section titled demographic effects, I don't think the word effects is appropriate, as this is an observational, cross-sectional study. A better word would be associations.
Discussion:

• Overall, this section overreaches mechanistically given the data, and it needs tighter alignment with what was actually tested. Large sections move quickly from a brief cross-sectional, retrospective self-report measure to specific neurocircuit claims and to a new construct. Those are interesting hypotheses, but the current wording sometimes reads like the data adjudicate mechanisms. I would recommend tightening this into a more disciplined interpretation: state clearly that the biological pathway discussion is speculative, keep causal language out, and emphasise plausible alternative explanations (shared method variance, reverse causality, mood-congruent recall).
• As this is a self reported measure across the lifespan, i.e. you are relying on recall of childhood sense of safety, the childhood perception of sense of safety which you collected for this study might be biased.
• In the section titled neurobiological pathways supporting safety, you refer to your study results as protective effects. I see that there may be longitudinal or other evidence referred to, but your study is not confirmatory for this. Overall, in this section, there needs to be a bit more of a relation back to your study's findings. How do your findings specifically contribute to this body of work? You also refer to a relationship between stress and cognitive aging. However, in your study, you excluded those that were experiencing cognitive decline.
• Key interpretive gaps are not fully addressed, especially the null depression finding and the "adult safety is stronger" pattern. The paper notes weak or null associations with depression and stronger effects for adulthood safety, but does not sufficiently unpack what those patterns could mean. You could strengthen this by offering a few concrete interpretations for the depression result, and acknowledging that stronger adulthood effects could reflect temporal proximity and recall processes rather than cumulative lifespan effects. Relatedly, the Kenya-specific implications would land better if you grounded "adult safety" in salient, concrete domains (e.g., gendered violence, economic insecurity, neighbourhood infrastructure, work precarity) rather than primarily in general theory.

Version 1:

Reviewer comments:

Reviewer #1

(Remarks to the Author)

Reviewer #2

(Remarks to the Author)
The authors have thoroughly addressed my comments. The revised manuscript is publication-ready.

Open Access This Peer Review File is licensed under a Creative Commons Attribution 4.0 International License, which

permits use, sharing, adaptation, distribution and reproduction in any medium or format, as long as you give appropriate credit to the original author(s) and the source, provide a link to the Creative Commons license, and indicate if changes were made.
In cases where reviewers are anonymous, credit should be given to 'Anonymous Referee' and the source.
The images or other third party material in this Peer Review File are included in the article's Creative Commons license, unless indicated otherwise in a credit line to the material. If material is not included in the article's Creative Commons license and your intended use is not permitted by statutory regulation or exceeds the permitted use, you will need to obtain permission directly from the copyright holder.
To view a copy of this license, visit https://creativecommons.org/licenses/by/4.0/

COMMSMED-26-0107A

Title: Safety as a Social Determinant of Brain Health in Kenya

Reviewer #1 (Remarks to the Author):

The paper presents two aims: to evaluate the Lifetime Sense of Safety scale's psychometric properties and to examine associations with established social determinants of brain health in a Kenyan sample. This is an important contribution, as validated measures of safety perception in low- and middle-income contexts are needed to advance research on social determinants of health. My comments are below.

1.1 The introduction does not clearly define what constitutes "brain health." Given that this is the central focus of the paper, a clear operational definition is essential.

Response:

This is a great suggestion that strengthens the conceptual foundation for the study. We have added an explicit operational definition of brain health to the Introduction, drawing on the World Health Organization's framework. We now specify that brain health refers to the preservation of cognitive capacities and psychological wellbeing that enables individuals to reach their full potential across the lifespan. This definition consistent with the WHO's global action plan on dementia prevention. Specifically, we have added this second paragraph to the Introduction:

"Brain health refers to the preservation of cognitive functioning and psychological wellbeing that enable individuals to realize their full potential and to adapt to stressors across the lifespan, consistent with the World Health Organization's global action plan on dementia prevention [Seeher 2025]. Operationalized in this way, brain health is a multidimensional construct that spans: (1) psychological wellbeing, as commonly indexed by depression and anxiety scales; (2) allostatic load, as commonly indexed by perceived stress and stressful life events; (3) protective capacity, as commonly indexed by measures of psychological resilience; and (4) cognitive function, as commonly indexed by objective memory performance."

1.2 The study employs a wide range of outcome variables, including the PHQ-9, stress scales, and CERAD word list, yet the extensive introduction does not adequately lay the groundwork for how these multiple outcome indicators map onto the construct of brain health. Further clarification on how these diverse measures collectively capture or relate to brain health would strengthen the conceptual framework of the study. Additionally, summary statistics for all outcome variables would help readers better understand their distributions within the sample.

Response:

We thank the reviewer for this important point. We have revised the Introduction to explicitly articulate how each category of outcome variable relates to brain health.
"The present study has two aims. The first is to examine the psychometric properties of the LSS, including its factor structure, internal consistency, and test-retest reliability, in a large sample of Kenyan adults. The second is to evaluate the scale's associations with established social determinants of health (sex, education level, and multidimensional poverty) and specific indicators of brain health, selected because each is theoretically linked to felt safety, including: (1) anxiety, perceived stress, and negative life events, selected as proximal indicators of threat reactivity and allostatic load [2,6]; (2) depression, selected as a contrasting indicator less tightly linked to threat processing, to probe the specificity of safety-relevant associations; (3) psychological resilience, selected because safety schemas are posited to function as cognitive resources that facilitate adaptive coping; and (4) objective memory performance, selected as a direct measure of brain health [49]. In keeping with the observational, cross-sectional design, these associations are evaluated as correlational rather than causal. In brief, we find that the six-item LSS has good psychometric properties in Kenyan adults, that it contains two correlated childhood and adulthood factors, and that higher LSS scores are associated with lower anxiety and stress, higher psychological resilience, and better objective memory, with consistently stronger associations for adulthood than childhood safety."
Summary statistics (means and standard deviations) for all outcome variables have been added to the Results section as Table 3.

1.3 Regarding the composite memory index, it is unclear which specific subtests were included, whether z-score standardisation was used, or how variability across subtests was handled. Additional details on these procedures would be helpful.

Response:
The composite memory index was derived from four subtests: CERAD word list total learning (trials 1-3 summed), CERAD delayed recall, Brave Man Story immediate recall, and Brave Man Story delayed recall. Raw scores for each subtest were converted to z-scores using the sample mean and standard deviation, and the four z-scores were then averaged to yield the composite index. This centering and averaging approach is standard for creating composite cognitive indices in aging research. We have now described these procedures in detail in the Measures section of the manuscript and provided summary statistics in Table 3.

1.4 The rationale for adjusting for education requires clarification, particularly given that education also appears to be treated as an outcome in the discussion. This dual role creates conceptual ambiguity that should be addressed.

Response:
Education was included as a covariate in the LSS-memory correlation to isolate the association between lifetime safety and memory performance from the well-established confounding influence of formal education on cognitive test performance. We do not treat education as an outcome of safety in our analyses, although we discuss the theoretically bidirectional relationship between education and safety in early life. In our analyses, we treat education as a known social determinant of health. This further supports the need to adjust for its confounding influence on the memory composite score.

1.5 Please provide detailed information regarding the cutoff criteria for fit indices used in the analysis section. It would be beneficial to include specifics about the methods employed in the Exploratory Factor Analysis (EFA), the statistical tests conducted, and the corresponding cutoff values.
Response:
The EFA was conducted using principal axis factoring (PAF) as the extraction method, which is appropriate for scales where items are assumed to share common variance due to underlying latent constructs. Factor retention was guided by parallel analysis (which supported a two-factor solution) and the scree plot criterion (inflection point after two factors). Oblimin rotation was used because the two factors were expected to be correlated, given the developmental continuity between childhood and adulthood safety. Criteria for adequate factor loadings were set at loading >= 0.40. The Kaiser-Meyer-Olkin (KMO) value of 0.82 meets the commonly cited threshold of >= 0.80 for 'meritorious' sampling adequacy (Kaiser, 1974). Bartlett's test of sphericity was significant (chi-square = 734, $p < 0.001$), confirming that the correlation matrix was factorable. These details have been added to the Methods/Analysis section.

1.6 The analysis appears to lack tables that present the factor loadings. Including these would greatly enhance the readability and comprehensiveness of the findings.
Response:
We agree and have added a table presenting the pattern matrix of factor loadings from the oblimin-rotated EFA, including loadings for each of the six items on both factors. This table is now included in the Results section (Table 2). Item loadings on the primary factor ranged from 0.83 to 0.90 for the three childhood items and from 0.74 to 0.92 for the three adulthood items, with minimal cross-loadings.

1.7 A distribution of the responses to the 10-point scale questions would be helpful for understanding the data more thoroughly.

Response:
We added histograms showing data distributions for each item (Figure 1B). Consistent with the reported negative skew (ranging from -0.78 to -1.10), item means ranged from 7.28 to 7.68, with a concentration of responses in the upper range of the scale (7-10), reflecting the medically healthy and psychiatrically healthy nature of the sample. These distributional characteristics are described in the psychometrics subsection of the Results and now in Figure 1B.

1.8 There are concerns regarding the use of Cronbach's alpha. Recent literature suggests that McDonald's omega may provide a more accurate and robust measure of reliability, and the authors should consider this alternative.
Response:
We agree that McDonald's omega (omega_total) is a sound estimate of reliability when the assumption of equal factor loadings is not met and have reported McDonald's omega alongside Cronbach's alpha. The omega value was 0.82, which is consistent with the alpha of 0.83 and similarly indicates good internal consistency. The modest difference between the two coefficients is expected given the relatively homogeneous factor loadings.

1.9 The rationale for employing EFA rather than Confirmatory Factor Analysis (CFA) requires further clarification, particularly given that the stated aim is validation. EFA is generally used to develop and explore new scales, whereas CFA is typically employed to validate the factor structure in a new or independent sample. The authors should address why EFA was deemed more appropriate for this study's objectives.
Response:
The decision to use EFA rather than CFA reflects the genuinely exploratory nature of the factor structure for this scale at this early stage of development and validation. While the item content was guided by theory (childhood versus adulthood settings), the LSS is a novel scale with no prior psychometric data and no previously validated factor structure to confirm. In this context, EFA allows the data to determine the latent structure empirically, without imposing a priori constraints that could lead to misspecification. CFA would be the appropriate next step in an independent sample (ideally one with different demographic characteristics or drawn from a different population) to formally confirm the two-factor structure identified here. We have explicitly acknowledged this distinction in both the Analysis section and in the Limitations section, and we have identified independent-sample CFA as a priority for future validation work.

1.10 The limitations section does not adequately address the inherent constraints and potential issues associated with using EFA. A discussion of these methodological limitations would strengthen the paper.

Response:

We agree that the Limitations section required expansion in this area. We have added the following: "Fourth, the two-factor solution was derived from exploratory factor analysis in a single sample and should be considered provisional pending confirmatory analysis independent samples."

Regarding the need for future research, we state: "confirmatory factor analysis and tests of measurement variance across sex, socioeconomic, and clinical groups are needed before between group comparisons can be interpretated with confidence."

1.11 The scale was tested in a clinical setting. The authors could discuss the implications for the use of the scale outside of clinical contexts, including its generalisability and applicability to community or population-based settings.

Response:

Our recruitment strategy requires further clarification. Although research procedures took place in a clinic setting, we purposively recruited participants from the larger community to reflect the demographic and socioeconomic composition of Nairobi County. Our strategy involved word-of-mouth, social media, and engaging community health promoters across different sub-counties. This is now more clearly communicated in the Methods. There were a proportionate number of males/females and people classified as multidimensionally poor compared with census data from Nairobi County, suggestive of a representative sex and socioeconomic distribution.

Reviewer #2 (Remarks to the Author):

This manuscript addresses an interesting and potentially important gap in the literature by positioning safety as a social determinant of brain health and by attempting to operationalize this construct in a Kenyan context. The focus is novel, the setting is highly valuable, and the effort to develop a brief, visually accessible measure that may be feasible across education levels is a clear strength. I also appreciated the authors' attempt to link perceived lifetime safety not only to psychological functioning, but also to cognitive outcomes, which gives the paper broader conceptual relevance for global mental health and cognitive aging research. Overall, this manuscript has the potential to make a meaningful contribution. However, there are several major conceptual, methodological, and interpretive issues that should be addressed before the manuscript is suitable for publication.

Overall:

2.1 Your scale relies on self-reported perception of safety in childhood, measured during adulthood, as well as self-reported measures of associated factors. I think this could bias your results, thus should be factored in to the conclusions.
Response:
We agree that self-report bias is an important factor that should be considered when interpreting results. In the Discussion, we strengthened the existing acknowledgment of retrospective recall limitations and added explicit language about shared method variance as potentially inflating correlations between LSS and psychological outcomes. We also point out findings that make it unlikely that results are due to recall bias alone
"Second, retrospective assessment of childhood safety may be subject to recall and mood biases, though moderate test-retest reliability suggests reasonable stability of subjective safety appraisals and the null association with depression argues against mood-congruent recall bias. Third, shared method variance from self-report can inflate correlations among psychological scales; however, the observed association between LSS scores and objective memory performance mitigates this concern."
We also call for future work to examine the relationship between LSS and biomarkers of accelerated aging, including inflammatory markers, cortisol dynamics, and neuroimaging indices of brain health, which would address this limitation.

2.2 Throughout the paper, causal language is applied to refer to study aims and results and conclusions. This should be modified to reflect the design of the study.
Response:
We have removed causal language. Key changes include: (1) replacing 'confers protection against' with 'is associated with' in the aims and analyses; (2) replacing 'protective effects' with 'associations' or 'correlates' throughout the Results and Discussion; (3) revising the abstract conclusion to remove the phrase 'protective effects' where it implies causation; (4) changing 'effects' to 'associations' in section headings (e.g., 'Demographic Effects' revised to 'Demographic Associations'); and (5) flagging neurobiological pathway discussion as speculative throughout the Discussion. We have been careful to retain the term 'protective' only where it is explicitly framed as a theoretical proposition or in reference to findings from longitudinal literature, not from the present data.

2.3 Sampling and generalizability are more limited than the framing implies. This is a clinic-based, largely urban Nairobi sample with an overrepresentation of higher education, plus exclusion of anyone self-endorsing common psychiatric

conditions. That combination likely truncates both exposure and outcome variability and may inflate or distort associations (for example, safety with anxiety and memory) relative to what you would see in more socioeconomically diverse community samples, rural settings, or among people with current mental health problems. I would strengthen the manuscript by being more explicit that this is an initial validation in a specific sampling frame, and by outlining a concrete next-step validation plan (for example, community sampling, rural counties, inclusion of people with depression or trauma histories, and replication of factor structure and correlates).

Response:

Although the setting where research was conducted was in a clinic, the sample recruitment strategy is not clinic-based. We worked with community health promoters (the term used in Kenya for community health volunteers) to implement a purposive community-based sampling strategy. This allowed us to recruit participants from different socioeconomic strata, including informal settlements. However, it is true that the sample was recruited from an urban region and may be truncated in outcome variability due to exclusion of clinical groups. In the Limitations section, we have expanded discussion around limits to generalizability and outlined a future validation plan including replication of the two-factor structure via CFA in independent samples and measurement invariance testing across different socioeconomic and clinical groups.

2.4 The psychometrics are promising, but key details and sensitivity checks are missing for a scale-validation paper. Right now, the results lean heavily on alpha, EFA, and a single test-retest correlation. I would expect: (a) fuller reporting of the EFA (extraction method, eigenvalues/scree, factor correlation, communalities, cross-loadings), (b) some assessment of measurement performance across education and sex (at minimum, invariance checks or a clear plan to test invariance), and (c) sensitivity analyses around the pro-rating/interpolation rules, since imputing school and work from flanking items may mechanically reinforce the two-factor structure and dampen missingness-related uncertainty. Even a short appendix showing results with and without interpolated cases would help.

Response:

We have substantially expanded the psychometric reporting in response to this comment and in response to overlapping concerns from Reviewer 1 (see Tables 2 and 3). We have added: (a) a report of eigenvalues, (b) the inter-factor correlation from the oblimin rotation; (c) sensitivity analyses for all bivariate associations with and without controlling for age, sex, and education (Table 3); and (d) a discussion of measurement invariance as a priority for future work. Regarding sensitivity analyses for the pro-rating rule: only 6 of 323 participants

(1.8%) required interpolation for school (N=4) or work (N=2) items, making a formal sensitivity analysis unlikely to alter findings meaningfully.

Abstract:

2.5 It would be good to have a bit more detail about the theoretical underpinning of this scale. How did you develop it? From where did you derive the items?
Response:
Although strict word count limits (150 words) for the Abstract preclude description of scale development, we have expanded the Methods section to describe the theoretical grounding of items in Social Safety Theory (Slavich, 2020), which posits that perceived safety across nested social contexts and accumulated lifespan stages calibrates stress systems and shapes long-term health. Accordingly, items were designed to capture perceived safety across proximal (home), institutional (school, work), and community (neighborhood) settings, and to distinguish childhood from adulthood experiences. Importantly, items were co-developed and reviewed for face validity in collaboration with Kenyan and Ugandan anthropologists, neuroscientists, and psychologists prior to piloting.

2.6 You mentioned you evaluated associations with established social determinants of brain health. If there is space, it would be good to highlight at least some examples of the factors you evaluated for association with safety.
Response:
We appreciate this observation as it has drawn our attention to the need for greater clarification of the social determinants of health that we assess (sex, education, multidimensional poverty). Although the strict word count of the Abstract precludes listing them all, we now clarify in the Methods/Measures section that these are the social determinants of health that we assess.

2.7 You mentioned that better memory was associated with psychological safety after adjusting for age and education. Were the other mentioned associations not adjusted for these factors? I think the methods should explicitly state the statistical tests conducted for deriving associations.
Response:
Age and education were included as covariates for the memory analysis because both are strongly and independently associated with cognitive test performance. However, your observation directed our attention to similar associations with these factors in the literature on anxiety, stress, depression, and resilience. In addition, it raised our awareness of the importance of sex as a covariate. All of the psychological constructs we measured share an association with age, education, and sex to varying degrees. Therefore, we substantially revised our

approach and examined associations between LSS and these factors (anxiety, stress, depression, resilience, memory) with and without adjustment for age, education, and sex (described in text and presented in Table 3). This strengthens confidence in the pattern of findings, which are robust to inclusion of these covariates.

2.8 You mention effect sizes. It would be good to specify what effects you are referring to. Also, there was no experiment done here, so I think you should use the language appropriate to the test conducted. I am also wondering if you mean that these effects were stronger regarding the association with psychological resilience and cognitive health.

Response:

We have removed the word 'effect(s)' from the abstract and manuscript, replacing it with 'associations' throughout, consistent with the observational, cross-sectional design.

2.9 In your conclusion you mentioned that the scale is short, but nowhere in the abstract is the length of the scale mentioned.

Response:

We have removed the term “short” from the Abstract.

2.10 In your conclusion, you use the word effects, but there was no experiment conducted in your study, so this language is inappropriate.

Response:

Agreed. We have replaced 'effects' with 'associations' to ensure consistent use of observational language throughout the Abstract and Manuscript.

Introduction:

2.11 You state that the LSS was developed to address measurement gaps which you outline in the previous paragraph. However, according to the current aim, it is unclear how your study addresses these measurement gaps other than the lifespan measurement. For instance, does it focus on aspects of safety other than trauma?

Response:

We have revised the Introduction to more clearly articulate the multiple measurement gaps addressed by the LSS: (1) existing scales assess only current safety perceptions, while the LSS captures retrospective lifespan safety; (2) existing tools focus on specific environments (e.g., neighborhood safety) or specific threats (e.g., trauma-focused ACE questionnaire), while the LSS

integrates multiple settings across two developmental periods; (3) existing psychometric tools have limited feasibility across low-literacy or low-resource settings, while the LSS uses a visual analog color bar to reduce literacy barriers.

2.12 The second aim of the study which seems to be about the association of your new measure with several psychosocial constructs needs clarification. How is this related to lifetime sense of safety? What is the theoretical and scientific underpinning of the assessment of LSS's association with these factors? How were they selected?

Response:

We have revised this section of the Introduction to ground the second aim in theory and to explain the selection logic for each outcome domain: "*The second aim is to evaluate the scale's associations with established social determinants of health (sex, education level, and multidimensional poverty) and specific indicators of brain health, selected because each is theoretically linked to felt safety, including: (1) anxiety, perceived stress, and negative life events, selected as proximal indicators of threat reactivity and allostatic load; (2) depression, selected as a contrasting indicator that is less tightly linked to threat processing, to probe the specificity of safety-relevant associations; (3) psychological resilience, selected because safety schemas are posited to function as cognitive resources that facilitate adaptive coping; and (4) objective memory performance, selected as a direct measure of brain health.*"

Methods:

2.13 From your description of the scale development, it is unclear how the items were derived and what domains are included. Please elaborate on how was it determined that this scale should be 6 items. Are there 6 domains of safety? Is there any theoretical basis for the selected scale items?

Response:

We responded to this concern above (see 2.5) but include again here for ease of reference. The Methods/Scale Development section was revised to briefly describe the theoretical grounding of items in Social Safety Theory (Slavich, 2020), which posits that perceived safety across nested social contexts and accumulated lifespan stages calibrates stress systems and shapes long-term health. Accordingly, items were designed to capture perceived safety across proximal (home), institutional (school, work), and community (neighborhood) settings, and to distinguish childhood from adulthood experiences. The use of six items was a pragmatic decision to keep the scale brief and feasible in multi-measure battery contexts. The item set was co-developed, reviewed, and refined by Kenyan social scientists, anthropologists, and clinicians, who agreed that

these six settings represented salient and culturally appropriate domains of safety in the Kenyan context.

2.14 Your inclusion criteria for languages seems to leave out other language speakers that don't speak Kiswahili or English, i.e. those who might not have gone through the education system, which you mention in paragraph 2 of the scale development section. This could potentially explain the fact that the general population's education level is different from your study's.

Response:

The requirement for fluency in Swahili or English, the two official languages of Kenya and the languages of instruction in formal education, could introduce selection bias toward individuals with some formal schooling but it should be noted that the proportion of people with no formal education in our sample is commensurate to the general population. Kenya has over 40 indigenous languages and it was not within the scope of this study to translate and pilot the scale across each of these languages. This limitation is now explicitly acknowledged, where we note that the language inclusion criterion limits generalizability and that future adaptations of the LSS in different languages is needed.

2.15 From how the results are currently written, it is not clear why you excluded those with certain health conditions. I imagine this was part of the aims of the larger studies. It seems this would bias your sample towards a significantly higher proportion of healthy people than the general population.

Response:

The exclusion of individuals with self-endorsed psychiatric, neurological, and medical conditions was an eligibility criterion of the two parent studies (AD-DETECT and BRK), both of which aimed to characterize cognitive aging in community-dwelling adults without pre-existing conditions that would confound cognitive outcomes. We discuss the implications of this more fully in the revised Limitations section. There are advantages to excluding adults with cognitive impairment or active psychiatric disorders in first-stage scale development, such as clean estimation of factor structure and reliability before extending validation to clinical populations where state-dependent variance might confound the psychometric properties. This is especially important in sub-Saharan African settings where large variation in educational attainment and socioeconomic resources need to be considered as covariates without the confounding influence of psychiatric or neurocognitive disorders.

2.16 In the analysis section, you claim that you assessed whether sense of safety confers protection against cognitive decline. However, as your study is observational and cross-sectional, you cannot assess this.

Response:

Agreed. The word 'confers' and the phrase 'protection against cognitive decline' have been removed from the Analysis section (and from equivalent passages in the Abstract and Results) and replaced with “associated with” to reflect the correlational nature of relationships in our cross-sectional data.

Results:

2.17 In the section titled demographic effects, I don’t think the word effects is appropriate, as this is an observational, cross-sectional study. A better word would be associations.

Response:

Agreed. The section heading has been changed to 'Demographic Associations' and the word 'effects' has been replaced with 'associations' throughout the Results section.

Discussion:

2.18 Overall, this section overreaches mechanistically given the data, and it needs tighter alignment with what was actually tested. Large sections move quickly from a brief cross-sectional, retrospective self-report measure to specific neurocircuit claims and to a new construct. Those are interesting hypotheses, but the current wording sometimes reads like the data adjudicate mechanisms. I would recommend tightening this into a more disciplined interpretation: state clearly that the biological pathway discussion is speculative, keep causal language out, and emphasise plausible alternative explanations (shared method variance, reverse causality, mood-congruent recall).

Response:

We have substantially revised the Discussion to clearly state how our cross-sectional data precludes causal or directional interpretation. We describe how our associational findings are consistent with results from a prospective 5-year longitudinal study linking subjective neighborhood safety to (prospective) stable cognitive trajectories in older adults. We make it clearer that the mechanistic animal and human studies described are meant to substantiate links between safety and neuroplasticity, as well as between safety and chronic stress/threat/allostatic load, as a means to contextualize our correlational findings

in the current literature on fear neurocircuitry and memory. However, we inserted statements to more clearly differentiate these mechanistic findings from our current correlational findings, and now state what would be needed in the future to link self-reported safety to neural substrates. We have added caveats to interpreting the null depression finding in a psychiatrically healthy sample. Finally, we added discussion of the way in which shared method variance and mood-congruent recall limit interpretation.

2.19 As this is a self reported measure across the lifespan, i.e. you are relying on recall of childhood sense of safety, the childhood perception of sense of safety which you collected for this study might be biased.

Response:

We agree, and have strengthened our treatment of this limitation in two ways. First, the Limitations section now explicitly identifies retrospective assessment of childhood safety as susceptible to recall bias. We note that the moderate test-retest reliability suggests stability of safety appraisals, that the association with objective memory performance reduces concerns that results are solely based on shared method variance, and that the null association between LSS and current depressive symptoms argues against mood-congruent bias. The expanded Future Research agenda now includes prospective longitudinal designs and triangulation of the LSS with biomarker and neuroimaging measures to address recall-bias concerns.

2.20 In the section titled neurobiological pathways supporting safety, you refer to your study results as protective effects. I see that there may be longitudinal or other evidence referred to, but your study is not confirmatory for this. Overall, in this section, there needs to be a bit more of a relation back to your study's findings. How do your findings specifically contribute to this body of work? You also refer to a relationship between stress and cognitive aging. However, in your study, you excluded those that were experiencing cognitive decline.

Response:

We have revised the Discussion to clarify that the term "protective" or "protectome" refers to a larger body of work, beyond the present study, that shows a positive relationship between specific exposures and good health outcomes. Our findings provide initial preliminary evidence that "safety" as a construct should be included in future studies of risk and protective factors on aging. The revised discussion now includes more emphasis on the preliminary nature of our scale validation. We provide empirical rationale for inclusion of safety in exposome research by referencing mechanistic work from outside our study. This is needed to substantiate

the call for valid measurement tools in humans, particularly in settings where there is large variation in education and socioecononomic resources, which (as we show) is associated with safety experiences. Our findings contribute to the existing body of work by providing the first steps needed to develop and validate such as scale. We have revised the discussion to be clearer about future steps that will be needed to further validate the scale horizontally across different languages, cultural, and clinical groups and vertically by linking LSS scores to neural, physiologic, and fluid-based biomarkers.

2.21 Key interpretive gaps are not fully addressed, especially the null depression finding and the "adult safety is stronger" pattern. The paper notes weak or null associations with depression and stronger effects for adulthood safety, but does not sufficiently unpack what those patterns could mean. You could strengthen this by offering a few concrete interpretations for the depression result, and acknowledging that stronger adulthood effects could reflect temporal proximity and recall processes rather than cumulative lifespan effects. Relatedly, the Kenya-specific implications would land better if you grounded "adult safety" in salient, concrete domains (e.g., gendered violence, economic insecurity, neighbourhood infrastructure, work precarity) rather than primarily in general theory.

Response:

The first paragraph of the Discussion acknowledges that stronger associations with adulthood sense of safety could reflect temporal proximity to outcomes. We explored associations between safety and social determinants of health that are relevant to the Kenyan context. As a Low- and Middle-Income Country (LMIC), Kenya has wide variation in educational attainment and multidimensional poverty, which is why we explicitly captured and explored these factors as relevant for lifespan sense of safety. Finally, we expanded discussion of the null depression finding to address the concern about insufficient unpacking of the null safety-mood relationship:

"Interestingly, we did not find a strong association between LSS and depression, despite negative correlations with anxiety and stress. This provides preliminary evidence for divergent validity in a single sample that suggests safety may specifically associate with stress regulation rather than mood regulation per se. The safety construct, as operationalized by the LSS, may be more closely related to threat-detection and threat-regulation systems, while depressive symptoms are shaped by a broader set of losses and biological vulnerabilities that the LSS was not designed to capture. However, it is also possible that the medically and

psychiatrically healthy nature of our adult sample may have truncated the upper end of depressive severity and limited statistical power to detect an association."
