# Supplementary material for: Development and validation of the lifetime sense of safety scale in Kenyan adults
Source: Commun Med (Lond). 2026 Jun 6;6:483. doi: 10.1038/s43856-026-01715-2 (PMC13575095; doi:10.1038/s43856-026-01715-2)
Supplement: Supplementary file 2 [file 43856_2026_1715_MOESM2_ESM.pdf]

Supplementary Note 1

Lifetime Sense of Safety Scale – English

Instructions

This scale includes 6 questions about the degree to which you have felt a general sense of safety at different times and places in your life.

- **Safety means feeling secure, protected, or without fear.**
- **Unsafety means feeling afraid, insecure, or threatened.**

For each question, please rate your sense of safety using the 10-point colour scale. The right side of the colour bar indicates a greater sense of safety. A score of 10 is the highest sense of safety. The left side of the colour bar indicates unsafety. A score of 0 is the lowest sense of safety. For each question, please say the number or point the place on the colour bar that best indicates your response.

	**Score**
1. In your childhood, how safe did you feel at home?	
2. In your childhood, how safe did you feel at school?	
3. In your childhood, how safe did you feel in your neighbourhood?	
4. Throughout your adulthood, how safe have you felt at home?	
5. Throughout your adulthood, how safe have you felt at work?	
6. Throughout your adulthood, how safe have you felt in your neighbourhood?	
**Total Score**	

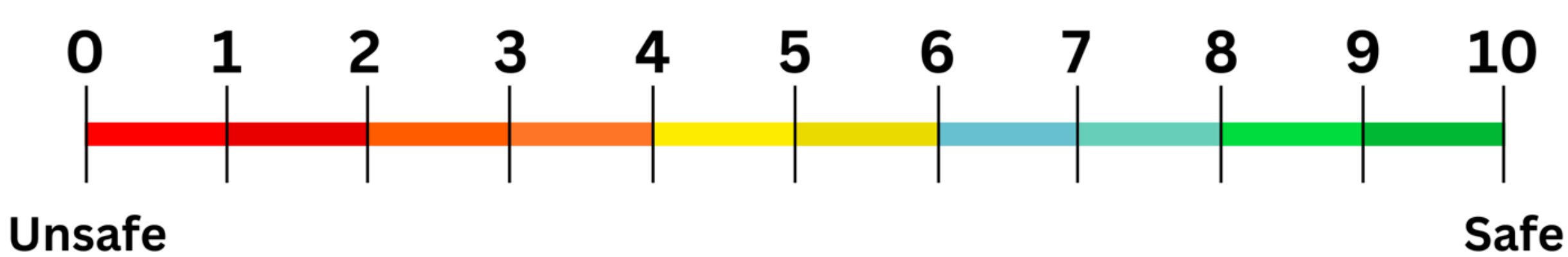

Lifetime Sense of Safety Scale – Swahili

Maagizo

Kipimo hiki kina jumuisha maswali 6 kuhusu kiwango ambacho umehisi usalama kwa jumla katika nyakati na maeneo tofauti maishani mwako.

- **Usalama unamaanisha kujihisi umelindwa, kukingwa, au bila uwoga**
- **Kutokuwa salama kunamaanisha kuhisi uwoga, kutolindwa, au kutishwa**

Kwa kila swali, tafadhali kadiria hali yako ya usalama kwa kutumia mizani ya rangi ya likert yenya alama 10. Upande wa kulia wa upau wa rangi unaonyesha hali ya usalama zaidi. Alama ya 10 ni hisia ya juu zaidi ya usalama. Upande wa kushoto wa upau wa rangi unaonyesha kutokuwa salama. Alama ya 0 ndio hisia ya chini zaidi ya usalama. Kwa kila swali, tafadhali sema nambari au uelekeze mahali kwenye upau wa rangi kuonyesha jibu lako vyema.

	**Alama**
1. Utotoni mwako, ulijihisi salama kiasi gani nyumbani?	
2. Utotoni mwako, ulijihisi salama kiasi gani shuleni?	
3. Utotoni mwako, ulijihisi salama kiasi gani katika ujirani/mtaa wako?	
4. Kwa maisha yako ya utu uzima, umejihisi salama kiasi gani nyumbani?	
5. Kwa maisha yako ya utu uzima, umejihisi salama kiasi gani kazini?	
6. Kwa maisha yako ya utu uzima, umejihisi salama kiasi gani mtaani mwako?	
**Jumla ya Alama**	

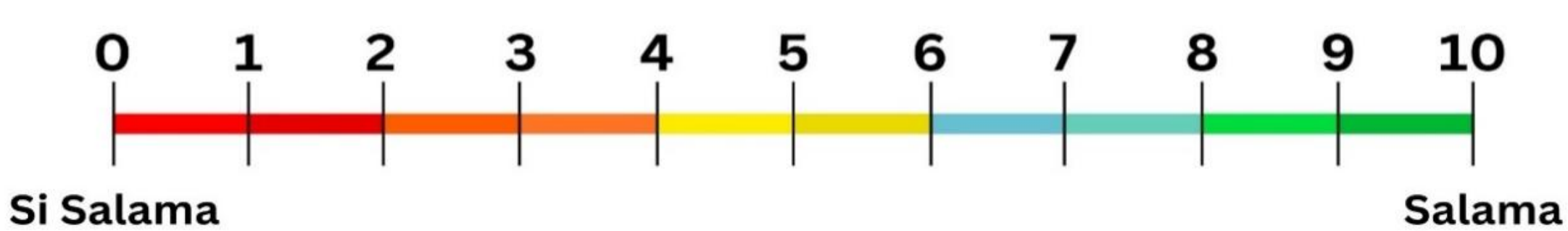

Administration and Scoring

- If person indicates that they did not attend school (#2), then put average from #1 and #3.

- If person indicates that they have not worked (#5), then put average from #4 and #6.

- If person indicates that they are not comfortable answering a question, ask if they are okay with pointing. If no, it is okay to skip a question or return to it later. The final score cannot be calculated if items #1, #3, #4, and #6 are not completed but participants should not be pressured to respond if they are not comfortable.

- If person indicates that they did not spend much time at work or school, explain that the quantity of time spent at school or work does not matter.

- If person indicates that they spent time in more than one home, school, workplace, or neighborhood, then ask them to respond for the one where they spent most of their time.

- Neighborhood can refer to anything outside the home or school, in the environment, and include places, people, animals, and climate/weather events.

- The total score should range from 0-60.
